# Intergroup contact in multiple adolescents’ contexts: The Intergroup Contact Interactions Scale (ICIS)

**DOI:** 10.3389/fpsyg.2022.1066146

**Published:** 2023-01-11

**Authors:** Savaş Karataş, Monica Rubini, Francesca Prati, Seth J. Schwartz, Elisabetta Crocetti

**Affiliations:** ^1^Department of Psychology, Alma Mater Studiorum University of Bologna, Bologna, Italy; ^2^Departments of Kinesiology, Health Education, and Educational Psychology, College of Education, The University of Texas at Austin, Austin, TX, United States

**Keywords:** intergroup contact, positive contact, negative contact, parental contact, adolescence, school, out-of-school contexts

## Abstract

In the present contribution, we aimed to test the psychometric properties of the Intergroup Contact Interactions Scale (ICIS). The ICIS is a tool that can easily be administered to assess ethnic minority and majority adolescents’ positive and negative intergroup contact in both school and out-of-school contexts. Study I included 169 adolescents in Italy (40.2% ethnic minority adolescents; 51.5% female; *M*_age_ = 14.41) and provided initial support for the two-factor structure (i.e., positive and negative contact) of the ICIS in both school and out-of-school contexts. Study II, conducted with a sample of 1,037 adolescents in Italy (26.5% ethnic minority adolescents; 59.7% female; *M*_age_ = 14.58), indicated that the fit of the two-factor ICIS structure was excellent for both school and out-of-school contexts. Measurement invariance across ethnic minority and majority adolescents was also established. Convergent validity was also ascertained by highlighting meaningful associations of adolescents’ positive and negative contact with the quantity of contact as well as with their perceptions regarding parents’ positive and negative contact with outgroup members. Study III, involving a sample of 641 adolescents in Turkey (32.9% ethnic minority adolescents; 69.6% female; *M*_age_ = 15.51), supported the two-factor structure, as well as convergent validity, of the ICIS in both contexts. Measurement invariance across ethnic groups was also established. Overall, these studies suggest that the ICIS is a reliable measure for studying positive and negative intergroup contact among ethnic minority and majority adolescents across school and out-of-school contexts.

## Introduction

International migration is increasing worldwide (International Organization for Migration, 2019). Despite its challenges (e.g., prejudice, ethnocentric and xenophobic tendencies; Crocetti et al., 2021), such a progressive increase in international migration brings out beneficial opportunities for intergroup contact among people with diverse ethnic and cultural backgrounds (Wagner et al., 2006; Schlueter and Wagner, 2008; Pettigrew et al., 2010). Contact between the members of migrant and host national groups becomes particularly important and salient during the developmental period of adolescence because young people expand their social relationships to include peers and develop their intergroup attitudes based on interactions with peers, especially those of diverse ethnic and cultural backgrounds (Feddes et al., 2009; Wölfer et al., 2016; Elias et al., 2021; Guan et al., 2022). These contact experiences among ethnic minority (i.e., individuals born outside the destination country or who have at least one parent born outside the destination country; European Commission, 2020) and ethnic majority adolescents can take place in several contexts, such as school (e.g., Schachner et al., 2015; Bohman and Miklikowska, 2020) and other important out-of-school contexts (e.g., neighborhood, sports club; Bekhuis et al., 2013; Merrilees et al., 2018). Notably, it is critically important to examine adolescents’ intergroup contact both at school (a relatively more structured context where young people spend a great deal of their time) and in out-of-school contexts that may involve relatively less structured contact interactions. As such, there is an urgent need for a valid and reliable scale that can be easily administered to adolescents to assess their intergroup contact across these contexts while maintaining its psychometric properties. Therefore, in the present studies, we aimed to test the psychometric properties of a short and age-appropriate scale to measure positive and negative valence of adolescents’ intergroup contact.

### Intergroup contact theory

In his seminal conceptualization of intergroup contact, Allport (1954) argued that contact between members of different groups, under the right conditions – i.e., cooperation and equal status of groups in a given context, as well as common goals and support from authorities − would lessen intergroup hostility and lead to more positive intergroup attitudes. A great deal of research has been devoted to testing the basic principles of Intergroup Contact Theory over the last six decades. A key meta-analysis on intergroup contact (Pettigrew and Tropp, 2006, 2008) indicated that, although there may be facilitating conditions that enhance the impact of contact (including Allport’s original optimal conditions), engaging in social contact (and particularly positive contact), in and of itself, has a clear and demonstrable positive impact on intergroup attitudes across different contexts.

Nevertheless, in outlining the optimal conditions for intergroup contact, attention has been devoted predominantly to positive valence of contact, even though intergroup contact experiences might also have negative valence (Barlow et al., 2012; Meleady and Forder, 2019; Schäfer et al., 2021). Whereas positive contact is characterized by warm, respectful, friendly, and pleasant interactions between members of different groups, negative contact refers to distant, insulting, intimidating, unfriendly, and unpleasant interactions with outgroup members (Hayward et al., 2017). A pivotal study considering both forms of intergroup contact (Barlow et al., 2012) contended that, although positive contact is more frequent, negative contact has a more substantial effect on prejudice and intergroup attitudes (i.e., positive–negative contact asymmetry; see also Graf et al., 2014; Techakesari et al., 2015). Yet, other studies have failed to provide strong evidence concerning such an asymmetry by suggesting equal and opposite influences of positive and negative contact vis-à-vis various intergroup outcomes (e.g., Visintin et al., 2016; Árnadóttir et al., 2018). However, further evidence is still needed to better understand the mutual dynamics of positive and negative contact, specifically in adolescence, because adolescents’ contact directly influences the development of their concurrent and prospective intergroup attitudes (Feddes et al., 2009; Ruck et al., 2011; Wölfer et al., 2016).

#### Positive and negative contact in adolescence

Most research (e.g., Bagci et al., 2014) with ethnic minority and majority adolescents has focused primarily on the beneficial effects of positive contact by examining cross-ethnic friendships – as one of the most potent forms of positive contact – (Pettigrew, 1998; see also Davies et al., 2011). Along this line, cross-ethnic friendships have been found to provide psychosocial benefits for adolescent development, such as developing more inclusive intergroup attitudes (Turner et al., 2007; Feddes et al., 2009; Wölfer et al., 2016), greater psychological and social well-being (Karataş et al., 2021), more favorable academic outcomes and school adjustment (Baysu et al., 2014; Bagci et al., 2017), together with an increased sense of safety and decreased sense of vulnerability (e.g., Munniksma and Juvonen, 2012; Graham et al., 2014).

Besides these findings, van Zalk et al. (2021) have recently focused on positive intergroup contact between ethnic minority (i.e., Asian British) and majority (i.e., White British) adolescents without referring specifically to cross-ethnic friendships. They found that adolescents’ positive contact in ethnically and culturally diverse schools increased positive attitudes toward outgroup members by reducing intergroup anxiety and improving their knowledge about outgroup willingness for contact. A similar finding, indicating the mediating effect of intergroup anxiety in the associations between positive contact and negative intergroup attitudes, also emerged in The Netherlands, although the effect was more substantial for Dutch majority adolescents than for Muslim minority adolescents. In terms of adolescents’ negative contact and their correlates, however, both direct or indirect (through intergroup anxiety) associations were found among majority, but not minority, adolescents (Vedder et al., 2017). Despite these latter findings, studies have found that immigrant adolescents tend to indicate less positive intergroup attitudes and a lower sense of belonging to the destination culture when they engage in more negative contact in the form of unequal treatment and discrimination (Kende et al., 2021). In addition to these additive effects of positive and negative contact, a recent study testing the specific interactions between positive and negative contact (Árnadóttir et al., 2022) has also suggested that negative contact may undermine, at least to some extent, the favorable effects of positive contact among ethnic minority youth. Taken together, these findings underscore that positive and negative contact represent distinct forms of intergroup interactions, and that both deserve attention.

As a result, adopting a context-oriented approach (looking at school and out-of-school contexts separately) could enable to better understand how positive and negative contact and their correlates might be driven by contextual factors. This is specifically relevant in adolescents, whereby developmental changes might be influenced by interactions within and across socialization contexts (Bronfenbrenner and Morris, 2006). Among the different adolescents’ contexts, school has been conceived as the primary context because youth spend most of their time there and most of the optimal conditions for positive contact (e.g., equal status of groups) are provided through supportive institutional diversity norms (Tropp et al., 2022; Karataş et al., 2023). Out-of-school contexts, on the other hand, could involve a variety of more or less normative and frequent contact situations for adolescents. Therefore, the effects of adolescents’ positive and negative contact on various intergroup outcomes might differ depending on whether their contact occurs in relatively more or less structured socialization contexts (i.e., school and out-of-school contexts, respectively).

Indeed, Bekhuis et al. (2013) have provided initial insights regarding the differential links of perceived ethnic distance with adolescents’ positive and negative contact across schools and out-of-school contexts. They found that the effects of positive and negative intergroup contact in class were equally strong, whereas both positive and negative contact at sport clubs were unrelated to prejudiced attitudes toward ethnic outgroup members. This evidence supports our prior contention that positive and negative contact should be studied by considering school and out-of-school contexts, with the aim of better understanding the additive or interactive effects of these two forms of contact in adolescence. To this end, it is of critical importance to measure adolescents’ positive and negative contact by employing an age-appropriate scale that can easily be adapted to both school and out-of-school contexts.

#### Measurement of positive and negative contact in adolescence

So far, different approaches have been used to explore adolescents’ intergroup contact. Of these approaches, Social Network Analysis (SNA) has been increasingly applied to evaluate adolescents’ intergroup contact (e.g., Titzmann, 2012; Wölfer et al., 2016). Under the heading of SNA, it is essential to highlight the distinction between sociocentric and egocentric networks (Clifton and Webster, 2017). Sociocentric networks consider the direct and indirect social relationships within the naturally existing social structure in a given social setting (e.g., school, classroom) and, thus, represent an accurate and comprehensive way to appraise adolescents’ social contacts (Clifton and Webster, 2017; see also O'Donnell et al., 2021). Notwithstanding these advantages, this technique is restricted to a closed network (e.g., a specific classroom); it is, therefore, less suitable for capturing simultaneous contact experiences across multiple contexts. Moreover, it is also susceptible to missing data because missing participants can completely change the social network structure (see Wölfer and Hewstone, 2017). Although most of these drawbacks might be eliminated through alternative egocentric networks, which is an approach to assessing the personal networks of a specific respondent, these networks are completely subjective, like self-reported measures (Clifton and Webster, 2017). Therefore, self-report measures may still represent an essential way for assessing adolescents’ intergroup contact, especially when gathering large samples within a small span of time necessary.

However, most previously applied self-report scales have been designed to measure the quality of adolescents’ intergroup contact by employing either single (e.g., Mähönen et al., 2011) or multiple items (e.g., Merrilees et al., 2018) but without separating positive and negative contact as distinct yet relevant forms of intergroup contact (for exceptions, see Bagci and Gungor, 2019; Reimer et al., 2021; Árnadóttir et al., 2022). For example, in their studies with adolescents in Finland, Mähönen et al. (2011) assessed the quality of intergroup contact by employing a single-item measure rated on a 5-point-Likert scale to determine whether or not adolescents’ intergroup contact was generally pleasant. Merrilees et al. (2018) longitudinally measured intergroup contact quality by assessing positive interactions with outgroup members (i.e., equal, pleasant, friendly, cooperative, close, and intimate), but did not measure negative interactions. However, the advancements in the relevant literature (e.g., Pettigrew, 2008; Barlow et al., 2012, 2019; Aberson, 2015; Hayward et al., 2017) propose treating positive and negative contact as separate constructs so as to facilitate deeper insights into the dynamics of intergroup interactions. Some recent studies have assessed positive and negative contact in adolescence by employing one item for each (e.g., Bagci and Gungor, 2019; Bagci et al., 2022). However, this approach might not fully capture the multifaceted nature of adolescents’ contact (for a similar discussion, see Árnadóttir et al., 2022). Hence, testing the psychometric properties of a self-report measure including multiple but similarly structured items capturing positive and negative contact across adolescents’ socialization contexts might facilitate a greater understanding of how intergroup contact occurs in the lives of ethnic minority and majority youth.

### Overview of the present studies

Considering that several contextual properties (e.g., the ethnic composition of classes and neighborhoods; Merrilees et al., 2018; Lessard et al., 2019) might influence adolescents’ intergroup relations, it is critical to design a scale that can be applied across different socialization contexts while preserving its psychometric properties. To this end, we aimed to test the psychometric properties of an age-appropriate multiple-item measure that distinguishes between positive and negative interactions with outgroup members, as recommended by recent research on intergroup contact (Árnadóttir et al., 2022). More precisely, to capture the adolescents’ positive and negative intergroup contact across their multiple socialization contexts (i.e., school and out-of-school), we tested whether the ICIS can be applied to ethnic minority and majority adolescents. Initially, items assessing positive and negative contact based on the particular interactions with the outgroup members were drawn from Hayward et al. (2017). In their study, Hayward and colleagues proposed 37 and 32 items to assess positive and negative contact, respectively. From this pool, we selected eight items (i.e., four items for positive contact and four items for negative contact) assessing frequent positive and negative intergroup contact interactions, which could also be paired in terms of their different valence (e.g., “They have been friendly toward you; They have been unfriendly toward you”). In addition to these items related to specific types of contact-based interactions, we retained two equivalent but opposite items (i.e., The experience you had with host-nationals [foreign people] was positive [negative]”) based on prior studies in which these single items have widely been used to assess positive and negative contact (e.g., Barlow et al., 2012; Techakesari et al., 2015; Reimer et al., 2021). In this way, we designed a measurement tool consisting of 10 items (five for positive contact and five for negative contact) that could easily be rated by adolescents to assess their positive and negative contact across school and out-of-school contexts and within various types of studies (e.g., using longitudinal or experimental designs). However, considering that most of these items have been used with adults either separately or in different combinations, it is strictly necessary to test whether the ICIS can be reliably applied to ethnic minority and majority adolescents. Hence, in this set of studies, we aimed to evaluate the validity and reliability of the ICIS to assess adolescents’ positive and negative contact in school and out-of-school contexts.

In Study I, we tested the factor structure of the ICIS in both school and out-of-school contexts using Exploratory Factor Analysis (EFA) with ethnic minority and majority adolescents in Italy. Building on the EFA results in Study I, we aimed to provide further evidence for the psychometric properties of scores generated by the ICIS in two different *national contexts* (Kohn, 1987), namely Italy and Turkey (i.e., Studies II and III, respectively). In particular, Italy is a nation in which ethnic minority adolescents are mainly second-generation immigrants with frequent interactions with outgroup members, whereas Turkey has been a primary destination country for first-generation Syrian refugee adolescents who have relatively less frequent experiences with ethnic majority peers. Considering the quite different ethnic compositions of these two countries (see also Karataş et al., 2020; Prati et al., 2021), further testing of the psychometric properties of the ICIS in these countries would allow us to assess the robustness of the present tool.

In Study II, we tested the construct validity of the ICIS in school and out-of-school contexts with a large sample of adolescents in Italy. Furthermore, we evaluated measurement invariance to provide empirical evidence for the extent to which the ICIS can be employed equally well to measure positive and negative contact among ethnic minority and majority adolescents in Italy. Thereafter, in line with the intergroup contact literature, which emphasizes that positive contact is more frequent than negative contact (Barlow et al., 2012; Graf et al., 2014), we aimed to test the convergent validity of the ICIS by examining the associations between the frequency of adolescents’ direct contact with outgroup members (i.e., quantity of intergroup contact; Barlow et al., 2012) and their positive and negative contact. In addition, consistent with intergenerational transmission processes (Degner and Dalege, 2013) that suggest potential links of parents’ positive and negative contact with corresponding contact experiences among their children (e.g., Bagci and Gungor, 2019), we also aimed to disentangle the associations of adolescents’ positive and negative contact in both contexts with their perceptions concerning their parents’ positive and negative contact.

In Study III, we examined the psychometric properties of the ICIS in the Turkish context. In doing so, we initially tested its factor structure with a sample including ethnic minority and majority adolescents in Turkey. We then tested ethnic measurement invariance (i.e., ethnic majority and minority adolescents) for the ICIS separately within school and out-of-school contexts. Finally, as in Study II, we examined the convergent validity of scale by investigating the associations of adolescents’ positive and negative contact with the quantity of their own intergroup contact, on the one hand, and, on the other hand, with positive and negative intergroup contact of their parents.

## Study I: Testing the ICIS in a pilot study

Given that most ICIS items have been mainly used with adults, in this study, we aimed to test the factorial structure of the ICIS with a pilot sample consisting of ethnic minority and majority adolescents in Italy.

### Method

#### Participants

Study I included 169 adolescents (51.5% female; *M*_age_ = 14.41, *SD*_age_ = 0.74; age range 13–18), of which 101 were ethnic majority (i.e., Italian) adolescents and 68 were ethnic minority adolescents living in Italy (61.8% were second-generation and 38.2% were first-generation immigrants). In terms of language fluency, ethnic minority adolescents self-reported their fluency in Italian on a scale from 0 to 10. The mean scores of the first-generation (*M* = 8.52, *SD* = 1.80) and second-generation (*M* = 9.41, *SD* = 0.83) immigrant adolescents indicated that all participants were fluent in Italian.

The majority of participants (79.9%) came from two-parent families, 19.5% indicated that their parents were separated or divorced, and 0.6% reported other family situations (e.g., one deceased parent). Fathers’ educational levels were as follows: 37.5% held less than a high school diploma, 42.9% held a high school diploma, and 19.6% held a university degree. Mothers’ educational levels were as follows: 21.9% held less than a high school diploma, 52.7% held a high school diploma, and 25.4% held a university degree.

#### Measures

After obtaining both active participants’ assent and parental consent, participants completed an online questionnaire including socio-demographic questions (e.g., age, gender, and birth country) and the ICIS. The ICIS was translated from English into Italian in three steps: (1) two Italian versions of the scale were generated separately by a member of the authors’ team and a research assistant in social psychology; (2) the two translations were compared to each other, and disagreements were discussed until consensus was reached; and (3) the final Italian version was back-translated into English by another researcher, and this back-translated English version was compared to the original English version (Hambleton, 1994; van de Vijver, 2001; see Žukauskienė et al., 2020, for another study utilizing a similar procedure). The complete list of items in Italian is presented in Supplementary Table S3.

##### Positive and negative contact

The ICIS was used to assess adolescents’ positive and negative contact in school and out-of-school contexts. Initially, adolescents were asked to think about their interactions with outgroup members in school (out-of-school) during the last 6 months in response to the following prompt: “The following questions are about interactions you may have had in school (out-of-school contexts) with people of foreign origin (Italian people). Now think about the interactions you had in the last 6 months at school (out-of-school contexts).” The school and out-of-school forms of the ICIS each consisted of 10 items (5 for positive contact and 5 for negative contact), scored on a response scale ranging from 1 (*never*) to 5 (*very often*).

### Results

We performed separate EFAs using principal components analysis with oblique (i.e., direct oblimin) rotation in SPSS to test the structural validity of ICIS scores in school and out-of-school contexts. Kaiser-Meyer-Olkin (KMO) measure of sampling adequacy (KMO = 0.871 and KMO = 0.911 for school and out-of-school contexts, respectively), as well as Bartlett’s sphericity test (*χ*^2^ [45] = 1341.227, *p* < 0.001 for school context, and *χ*^2^ [45] = 1873.700, *p* < 0.001 for out-of-school contexts), were satisfactory to test ICIS in both contexts. The results of the EFA indicated two factors with eigenvalues greater than 1.00 for the ICIS in each context. The total variance explained for school and out-of-school contexts was 78.1% and 85.9%, respectively. Hence, we retained a two-factor solution for the ICIS in both contexts.

As for the ICIS in school context, the first factor (i.e., positive contact), consisting of five items, explained 50.3% of the total variability among the item responses, and the second factor (i.e., negative contact) consisting of the remaining five items explained 27.8% of the total variability among the item responses. Likewise, the first and second factors (i.e., positive and negative contact with five items per each) for ICIS responses in out-of-school contexts explained 59.6% and 26.3% of the total variance, respectively. As reported in Table 1, the factor loadings for the two-factor solution ranged from 0.750 to 0.944 and from 0.869 to 0.982 for intergroup contact interactions in school and out-of-school contexts, respectively. Pearson correlation coefficients indicated that the mean scores for adolescents’ positive and negative contact were negatively interrelated in school (*r* = −0.291, *p* < 0.001) and out-of-school contexts (*r* = −0.365, *p* < 0.001). Item-total correlations are also reported in Supplementary Table S1.

**Table 1:** Factor loadings of the ICIS across school and out-of-school contexts in Study I (pilot study in Italy).

Item number	School context		Out-of-school contexts	
	Positive contact	Negative contact	Positive contact	Negative contact
Item 1	**0.089**	0.084	**0.093**	0.051
Item 2	**0.944**	−0.002	**0.928**	−0.061
Item 3	**0.887**	−0.088	**0.953**	−0.040
Item 4	**0.943**	0.093	**0.982**	0.083
Item 5	**0.075**	−0.133	**0.869**	−0.066
Item 6	−0.054	**0.881**	−0.048	**0.891**
Item 7	0.003	**0.859**	0.022	**0.942**
Item 8	0.013	**0.909**	−0.050	**0.907**
Item 9	−0.010	**0.084**	0.032	**0.923**
Item 10	0.033	**0.888**	0.024	**0.913**

Bold indicates the highest factor loadings.

### Brief discussion for Study I

Overall, this pilot study provided preliminary support for the two-factor structure (i.e., positive and negative contact) of the ICIS in both school and out-of-school contexts. To confirm the EFA results, additional studies were conducted to test the psychometric properties of this instrument in the additional samples of adolescents in Italy (Study II) and Turkey (Study III).

## Study II: Testing psychometric properties of the ICIS in a larger sample of adolescents in Italy

Building upon the promising results from Study I, in Study II, we sought to further test the construct validity of the ICIS with a large sample of adolescents living in Italy. First, we examined the factor structure in both school and out-of-school contexts. Second, we tested measurement invariance across ethnic minority and majority adolescents. Third, we tested the convergent validity of the ICIS by examining the associations of adolescents’ positive and negative contact with the frequency of their contact in school and out-of-school contexts. In line with the previous findings suggesting that positive contact is usually experienced more frequently than negative contact (e.g., Barlow et al., 2012; Graf et al., 2014), we expected that quantity of contact would be positively related to positive contact across both contexts. Given that experiencing negative contact with the outgroup members triggers avoidance of further contact (e.g., Meleady and Forder, 2019; Bagci et al., 2022), we also hypothesized that quantity of contact would be inversely linked to negative contact in school and out-of-school contexts. Moreover, in accordance with previous literature indicating the correspondence between the parents’ and their children’s contact experiences (e.g., Bagci and Gungor, 2019; Karataş et al., 2021), we expected to find positive associations of adolescents’ positive and negative contact with their perceptions regarding positive and negative contact experienced by their parents.

### Method

#### Participants

Study II included 1,037 adolescents (59.7% female; *M*_age_ = 14.58, *SD*_age_ = 0.67; age range 14–17), of whom 762 were majority Italian adolescents and 275 were ethnic minority adolescents. Participants were attending the first year of secondary high schools located in the North-East of Italy. Regarding family structure, most participants (69.0%) indicated that they came from two-parent families, 23.3% reported that their parents were separated or divorced, and 7.7% indicated other family situations (e.g., one deceased parent). Almost all participants (97.5%) were living with one or both parents. Parents’ educational levels were as follows: among fathers, 37.4% held less than a high school diploma, 50.2% held a high school diploma, and 12.4% held a university degree; 28.0% of mothers held less than a high school diploma, 49.5% held a high school diploma, and 22.5% held a university degree.

With respect to the demographic characteristics of ethnic minority adolescents, 72.0% of these youth were second-generation immigrants, and the remainder were first-generation immigrants who had been living in Italy for an average of 7.64 years (*SD* = 5.21) at the time of data collection. As for the language fluency, both first-generation (*M* = 8.28, *SD* = 2.27, range 0–10) and second-generation immigrants (*M* = 9.28 *SD* = 1.06, range 0–10) were fluent in Italian. Most first-generation immigrants (67.5%) were born in other European countries, with Albanians, Moldavians, and Romanians as the most highly represented groups. Similarly, the majority of participants’ parents migrated from other European countries (45.4% and 56.6% of fathers and mothers, respectively), with Romanians and Albanians as the most represented groups. Other families migrated from Africa (18.8% and 17.9% of fathers and mothers, respectively), Asia (5.2% and 6.2% of fathers and mothers, respectively), South, North, and Central America (3.6% of fathers, 5.1% of mothers), and the Middle East (1.1% of fathers, 0.4% of mothers). In terms of reasons for migration, the majority of the participants indicated that their parents migrated for economic reasons (40.0% and 33.1% of their fathers and mothers, respectively) and family reunification (7.6% and 24.4% of their fathers and mothers, respectively), and the remainder either reported other reasons (e.g., study) or did not provide an answer to this question.

#### Procedure

Before initiating the study, we sought permission from school principals to administer the questionnaire at school. Researchers then contacted adolescents to inform them about the study and to ask for their active assent to participate. Participants received oral and written information about the study and were asked to sign the informed consent form. In addition to active youth assent, parental consent was also obtained. Data were collected in May 2019 through a paper-and-pencil questionnaire in adolescents’ classrooms during regular school hours.

#### Measures

Adolescents initially completed the Italian versions of the scales aimed at assessing positive and negative forms of contact. We further asked about adolescents’ perceptions regarding the frequency of their own contact with ethnic outgroup members (i.e., the quantity of intergroup contact) and their parents’ positive and negative contact.

##### Adolescents’ positive and negative contact

The ICIS was employed to assess positive and negative contact among ethnic minority and majority adolescents across school and out-of-school contexts (see Supplementary Table S3).

##### Quantity of intergroup contact

The frequency of adolescents’ contact in school and out-of-school contexts was measured by using two items (“In the past 6 months, have you met and talked with Italian people [foreign people] at school [out-of-school contexts]?”). These items were answered on a 5-point rating scale (1 = *never*, 5 = *very often*).

##### Parental positive and negative contact

Adolescents’ perceptions regarding their parents’ positive and negative contact were measured using two items (see Bagci and Gungor, 2019), one item for positive contact and one another for negative contact (i.e., “How frequently do your parents have positive [negative] contact with people from other ethnic groups?”), that were scored on a 5-point rating scale (1 = *never*, 5 = *very often*).

### Results

#### Preliminary analyses

We initially conducted missing value analyses. Rates of missingness varied between 4.1% to 6.0% across the items. Little (1988) missing completely at random (MCAR) test yielded a significant result, *χ*^2^ (830) = 1632.610, *p* < 0.001. However, the normed *χ*^2^, which can be used to correct the sensitivity of the *χ*^2^ to sample size (Bollen, 1989), was 1.96. This normed value suggests that our data were very likely missing at random. For this reason, all participants were included in the analyses, and missing data were handled using the Full Information Maximum Likelihood (FIML) procedure available in M*plus* (Muthén and Muthén, 1998-2017; Kelloway, 2015). Means, standard deviations, and Cronbach’s alphas are displayed in Table 2.

**Table 2:** Means (M), standard deviations (SD), and Cronbach’s alphas (α) of study variables in each study.

	Study I (pilot study in Italy)			Study II (in Italy)			Study III (in Turkey)		
	*M*	*SD*	α	*M*	*SD*	α	*M*	*SD*	α
1. Quantity of contact in school context				4.01	1.04		3.31	1.16	
2. Positive contact in school context	4.04	0.82	0.93	4.07	0.77	0.09	3.09	0.99	0.88
3. Negative contact in school context	1.69	0.82	0.92	1.64	0.71	0.84	2.28	1.00	0.85
4. Quantity of contact in out-of-school contexts				3.53	1.26		2.84	1.34	
5. Positive contact in out-of-school contexts	3.78	0.99	0.96	3.81	1.02	0.95	3.09	1.15	0.93
6. Negative contact in out-of-school contexts	1.73	0.87	0.95	1.63	0.77	0.09	2.07	1.02	0.89
7. Parents’ positive contact				3.03	1.22		3.16	1.16	
8. Parents’ negative contact				1.96	1.01		2.03	1.09	

#### Main analyses

**Figure 1:**
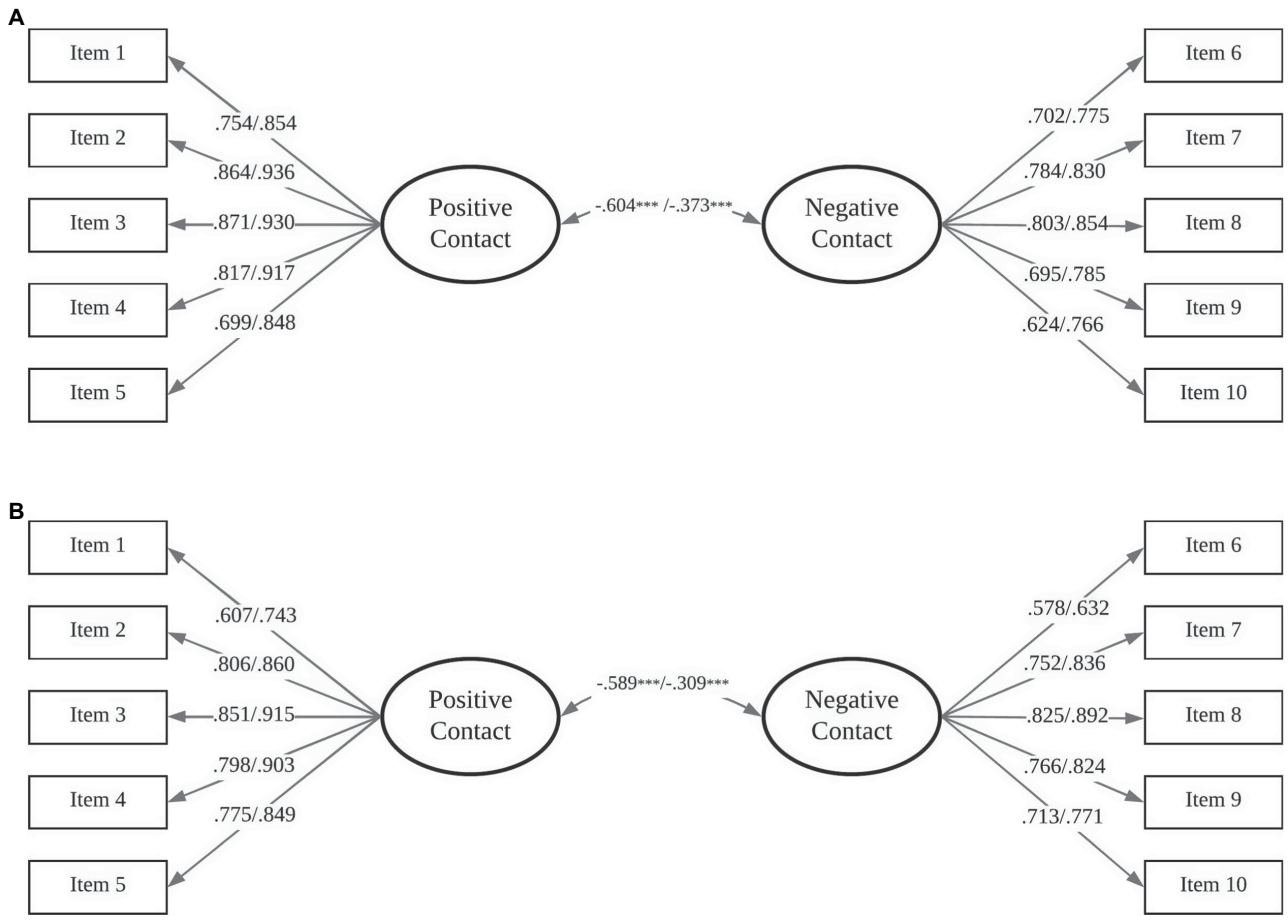
Factor loadings for the ICIS in Studies II **(A)**, and III **(B)**. The values presented on the left side of the slash marks indicate the standardized factor loadings of the positive and negative contact in school context, whereas the values displayed on the right side of the slash marks indicate the standardized factor loadings of the positive and negative contact in out-of-school contexts. All factor loadings and correlations are significant at *p* < 0.001.

To test the structural validity of the ICIS, we conducted CFAs in M*plus* using the maximum likelihood estimator with robust standard errors (MLR; Satorra and Bentler, 2001). We evaluated a solution with two latent variables (i.e., positive and negative contact) and 10 observed variables (i.e., five indicators for each latent variable) for both school and out-of-school contexts. Model fit was evaluated using the following criteria: The Comparative Fit Index (CFI) and Tucker-Lewis Index (TLI), with values higher than 0.90 indicating acceptable fit, and values higher than 0.95 demonstrating excellent fit; the Standardized Root Mean Square Residual (SRMR) and Root Mean Square Error of Approximation (RMSEA) with values below 0.08 representing acceptable fit and values lower than 0.05 suggesting excellent fit (Byrne, 2012). Moreover, the 90% Confidence Interval (CI) for the RMSEA was also examined (i.e., a good fit is indicated by an upper bound lower than 0.10; Chen et al., 2008). The CFA results (see Table 3) indicated an excellent fit for the two-factor model in both contexts. Standardized factor loadings (see Figure 1A) ranged from 0.624 to 0.871 and from 0.766 to 0.936 for intergroup contact interactions in school and out-of-school contexts, respectively. Negative correlations emerged between positive and negative contact in school (*r* = −0.604, *p* < 0.001) and out-of-school contexts (*r* = −0.373, *p* < 0.001). Item-total correlations are presented in Supplementary Table S1.

**Table 3:** Fit indices of the confirmatory factor analyses in Studies II and III.

	*χ* ^2^	*df*	CFI	TLI	SRMR	RMSEA [90% CI]
**Study II (in Italy)**						
The ICIS in school context	161.933	34	0.953	0.938	0.044	0.062 [0.052, 0.071]
The ICIS in out-of-school contexts	84.051	34	0.987	0.983	0.003	0.039 [0.028, 0.049]
**Study III (in Turkey)**						
The ICIS in school context	148.867	34	0.949	0.933	0.035	0.073 [0.061, 0.085]
The ICIS in out-of-school contexts	131.018	34	0.964	0.953	0.024	0.067 [0.055, 0.079]

*χ*^2^, Chi-square; *df*, degrees of freedom; CFI, Comparative Fit Index; TLI, Tucker-Lewis Index; SRMR, Standardized Root Mean Square Residual; RMSEA [90% CI], Root Mean Square Error of Approximation and 90% Confidence Interval.

#### Measurement invariance

To provide empirical evidence as to whether the ICIS can be applied equally well to assess positive and negative intergroup contact among ethnic minority and majority adolescents, three nested levels of measurement invariance were tested (Chen, 2007; Little et al., 2007; van de Schoot et al., 2012): (a) configural invariance, which requires that the same number of factors and pattern of fixed and freely estimated parameters hold across groups; (b) metric invariance, which indicates the equivalence of factor loadings and emphasizes that respondents from multiple groups attribute the same meaning to the latent construct of interest; and (c) scalar invariance, which implies the equivalence of both factor loadings and item intercepts and indicates that the meaning of the construct and the levels of the underlying items are equal across groups.

To statistically accept the assumption of metric or scalar invariance, at least two of three criteria must be satisfied: non-significant Δ*χ*^2^_SB_ (Satorra and Bentler, 2001), ΔCFI ≤ −0.010, and ΔRMSEA ≤ 0.015 (Chen, 2007). Results of measurement invariance tests (see Table 4) clearly suggested the presence of configural, metric, and scalar invariance for ICIS in both school and out-of-school contexts. Therefore, this instrument can be utilized to compare positive and negative contact among ethnic minority and majority adolescents. Latent mean comparisons indicated that ethnic minority adolescents reported significantly higher positive contact in both school (*p =* 0.011, Cohen’s *d* [95% CI] = 0.19 [0.05, 0.34]) and out-of-school contexts (*p* = 0.000, Cohen’s *d* [95% CI] = 0.62 [0.47, 0.77]), and higher negative contact in the school context (*p* = 0.019, Cohen’s *d* [95% CI] = 0.22 [0.08, 0.37]), compared to ethnic majority adolescents.

**Table 4:** Measurement invariance tests of the ICIS in school and out-of-school contexts in Studies II and III.

	Model fit indices							Model comparison				
	*χ*_SB_^2^	*df*	CFI	TLI	SRMR	RMSEA [90% CI]	Models	Δχ_SB_^2^	Δ*df*	*p*	ΔCFI	ΔRMSEA
**Study II (in Italy)**												
*Ethnic invariance of the ICIS in school context*												
M1. Configural model	209.788	68	0.949	0.933	0.046	0.065 [0.055, 0.075]						
M2. Metric model	223.224	76	0.947	0.938	0.054	0.062 [0.053, 0.072]	M2-M1	10.289	8	0.245	−0.002	−0.003
M3. Scalar model	252.335	84	0.094	0.936	0.055	0.063 [0.055, 0.073]	M3-M2	32.262	8	0.000	−0.007	0.001
*Ethnic invariance of the ICIS in out-of-school contexts*												
M1. Configural model	119.507	68	0.987	0.983	0.031	0.039 [0.027, 0.051]						
M2. Metric model	140.301	76	0.984	0.098	0.061	0.042 [0.031, 0.052]	M2-M1	22.811	8	0.004	−0.003	0.003
M3. Scalar model	166.081	84	0.979	0.977	0.064	0.045 [0.035, 0.055]	M3-M2	32.928	8	0.000	−0.005	0.003
**Study III (in Turkey)**												
*Ethnic invariance of the ICIS in school context*												
M1. Configural model	204.459	68	0.942	0.924	0.043	0.079 [0.067, 0.092]						
M2. Metric model	221.584	76	0.938	0.927	0.055	0.078 [0.066, 0.090]	M2-M1	15.91	8	0.044	−0.004	−0.001
M3. Scalar model	333.368	84	0.894	0.887	0.064	0.097 [0.086, 0.108]	M3-M2	119.781	8	0.000	−0.044	0.019
M3a. Partial scalar model^a^	239.985	80	0.932	0.924	0.056	0.079 [0.068, 0.091]	M3a-M2	19.772	4	0.001	−0.006	0.001
*Ethnic invariance of the ICIS in out-of-school contexts*												
M1. Configural model	178.779	68	0.096	0.948	0.031	0.072 [0.059, 0.084]						
M2. Metric model	192.859	76	0.958	0.095	0.004	0.070 [0.057, 0.082]	M2-M1	12.112	8	0.146	−0.002	−0.002
M3. Scalar model	258.213	84	0.938	0.933	0.054	0.081 [0.070, 0.092]	M3-M2	75.799	8	0.000	−0.002	0.011
M3a. Partial scalar model^a^	221.284	82	0.095	0.945	0.046	0.073 [0.062, 0.085]	M3a-M2	32.448	6	0.000	−0.008	0.003

*χ*_SB_^2^, Satorra–Bentler scaled Chi-square; *df*, degrees of freedom; CFI, Comparative Fit Index; TLI, Tucker-Lewis Index; SRMR, Standardized Root Mean Square Residual; RMSEA [90% CI], Root Mean Square Error of Approximation and 90% Confidence Interval; Δ = Change in the parameter. ^a^Intercepts of items 1, 6, 9, and 10 were released for intergroup contact in schools, whereas intercepts of items 1 and 9 were released for intergroup contact in out-of-school contexts.

#### Convergent validity

As reported in Table 5, bivariate correlations indicated that the quantity of contact that adolescents report in school context was positively correlated with their positive contact (*r* = 0.417, *p < *0.001) and weakly negatively correlated with their negative contact (*r* = −0.079, *p < 0.*05) in schools. Similarly, quantity of contact in out-of-school contexts was positively correlated with positive contact in the same context (*r* = 0.610, *p < 0.*001), and was unrelated to negative contact (*r* = −0.030, *p = 0.*351). Regarding the inter-context correlations, the relationship between positive contact in school and out-of-school contexts was large and highly significant (*r* = 0.494, *p* < 0.001). Likewise, a large positive correlation was found between adolescents’ negative contact in school and out-of-school contexts (*r* = 0.605, *p* < 0.001).

**Table 5:** Bivariate correlations among variables in studies II (in Italy) and III (in Turkey).

	1	2	3	4	5	6	7	8
1. Quantity of contact in school context	−	0.539^***^	−0.173^***^	0.348^***^	0.256^***^	−0.001	0.174^***^	0.023
2. Positive contact in school context	0.417^***^	−	−0.490^***^	0.302^***^	0.486^***^	−0.260^***^	0.234^***^	−0.061
3. Negative contact in school context	−0.079^*^	−0.540^***^	−	−0.097^*^	−0.258^***^	0.594^***^	−0.118^**^	0.169^***^
4. Quantity of contact in out-of-school contexts	0.445^***^	0.264^***^	0.000	−	0.632^***^	−0.083^*^	0.228^***^	0.040
5. Positive contact in out-of-school contexts	0.332^***^	0.494^***^	−0.256^***^	0.610^***^	−	−0.277^***^	0.255^***^	−0.066
6. Negative contact in out-of-school contexts	−0.046	−0.326^***^	0.605^***^	−0.030	−0.349^***^	−	−0.126^**^	0.165^***^
7. Parents’ positive contact	0.346^***^	0.309^***^	−0.141^***^	0.407^***^	0.423^***^	−0.178^***^	−	0.088^*^
8. Parents’ negative contact	−0.071^*^	−0.218^***^	0.338^***^	0.052	−0.091^**^	0.313^***^	−0.017	−

Bivariate correlations for Study II (in Italy) are presented below the diagonal, and bivariate correlations for Study III (in Turkey) are presented above the diagonal. *p < 0.05, **p < 0.01, ***p < 0.001.

In addition, as expected (see Table 5), parents’ positive contact was positively correlated with adolescents’ positive contact in both school (*r* = 0.309, *p < 0.*001) and out-of-school contexts (*r* = 0.423, *p < 0.*001). Similarly, parents’ negative contact was also positively associated with adolescents’ negative contact across contexts (*r* = 0.338, *p < 0.*001 and *r* = 0.313, *p < 0.*001, for school and out-of-school contexts, respectively). These findings suggest the correspondence between parents’ and adolescents’ contact. It should be noted, however, that adolescents reported both on their own contact and on their parents’ contact.

#### Mean differences

We then conducted a repeated measures ANOVA with two within-subject factors, namely contact valence (i.e., positive and negative) and context of contact (i.e., school and out-of-school). This analysis produced a significant interaction effect between the context and valence of adolescents’ intergroup contact, *F* (1, 975) = 31.651, *p* < 0.001, *η*^2^ = 0.031. That is, the context in which contact occurs appears to exert differential effects on the valence of adolescents’ contact (Table 2). Pairwise comparisons indicated that positive contact experiences were significantly more common in school versus out-of-school contexts. In contrast, mean scores for negative contact in school and out-of-school contexts did not significantly differ. Moreover, significant differences also emerged between mean scores of adolescents’ positive and negative contact in both contexts, suggesting that positive contact is more common than negative contact. Finally, a repeated measures ANOVA with contact quantity indicated that adolescents had more intergroup contact experiences in school than in out-of-school contexts, *F* (1, 990) = 214.905, *p* < 0.001, *η*^2^ = 0.178.

### Brief discussion for Study II

Overall, consistent with the EFA results from Study I, CFA results from Study II suggested that the two-factor model for positive and negative contact in school and out-of-school contexts fit the data very well. Results from Study II also indicated that the measure could be applied both to ethnic minority and majority adolescents to reliably measure their positive and negative contacts across both contexts. Findings also suggested that positive contact is more common than negative contact (e.g., Pettigrew, 2008; Barlow et al., 2012) and that adolescents’ own contact appears to be related to their perceptions of their parents’ intergroup contact (e.g., Bagci and Gungor, 2019). Finally, established mean differences for contact quantity and positive contact across school and out-of-school contexts further emphasized the necessity of adopting a context-oriented approach to gain a deeper understanding of adolescents’ contact. Even though all these findings together highlighted the robustness of scores generated by the ICIS in a larger group of adolescents from diverse ethnic and cultural backgrounds in Italy, further testing the psychometric properties of the ICIS in another cultural setting would be beneficial to provide more substantial evidence for its validity and reliability. Therefore, in Study III, the psychometric properties of the ICIS were examined in Turkey.

## Study III: Testing psychometric properties of the ICIS in Turkey

The main goal of Study III was threefold. The primary aim was to test the factor structure of the ICIS in the Turkish context with a sample of ethnic majority (i.e., Turkish) adolescents and their ethnic minority peers (primarily Syrian refugees). The secondary aim of this study was to examine the ethnic (i.e., Turkish adolescents versus ethnic minority adolescents) measurement invariance of the ICIS in both school and out-of-school contexts separately. The third aim was to examine the convergent validity of the ICIS in the Turkish context. As in Study II, we hypothesized that the quantity of contact would be positively related to positive contact and adversely linked to negative contact. Furthermore, we also expected that adolescents’ perceptions of parents’ positive and negative contact would be positively related to their children’s corresponding contact experiences.

### Method

#### Participants

This study included 641 adolescents (69.6% female; *M*_age_ = 15.51, *SD*_age_ = 0.84; age range 13–18), of whom 430 were ethnic majority adolescents (i.e., Turkish) and 211 were ethnic minority adolescents. Participants were attending the first or second years of different high schools in a large metropolitan area with more than two million inhabitants in the Southeastern Anatolia Region of Turkey. In terms of family structure, the majority of participants (92.0%) reported that their parents were married, 4.1% specified other family situations (e.g., one deceased parent), and 3.9% indicated that their parents were separated or divorced. Almost all participants (99.0%) indicated that they were living with at least one parent. The educational levels of participants’ parents were as follows: among fathers, 50.7% held less than a high school diploma, 28.9% held a high school diploma, and 20.4% held a university degree. Among mothers, 69.4% held less than a high school diploma, 21.5% held a high school diploma, and 9.1% held a university degree. Both fathers (*χ*^2^(2) = 103.612, *p* < 0.001) and mothers (*χ*^2^(2) = 134.569, *p* < 0.001) of ethnic minority adolescents were more highly educated than those of Turkish adolescents.

Regarding the demographic backgrounds of ethnic minority adolescents, 98.1% of these youth were first-generation immigrants. Of these first-generation immigrants, 98.6% were born either in Syria or have at least one parent born in Syria. The remaining three participants and their parents were born in other countries in the Middle East (i.e., Iraq, Saudi Arabia) and Africa (i.e., Egypt). On average, adolescents had been in Turkey for 4.59 years (*SD* = 1.60) at the time of data collection, and 38.9% of them had not visited their home country since their arrival in Turkey. Most participants reported that their parents migrated to Turkey between 2011 and 2019 to escape the war or to avoid serious political or economic difficulties (68.7% and 76.3% of their fathers and mothers, respectively).

#### Procedure

Following the same procedure used in Study II, the data collection was completed in April 2019 using a paper-and-pencil questionnaire in the classrooms during regular school hours.

#### Measures

As in Study II, all adolescents completed the same questionnaire. Given that the measures assessing adolescents’ perceptions of parents’ positive and negative intergroup contact were already available in Turkish (Bagci and Gungor, 2019), the remaining measures (the ICIS and the measure of quantity of contact) were translated from English into Turkish in three steps. First, three independent Turkish versions of the questionnaire were created by one of the authors and by two other bilingual researchers. Second, the three translations were compared to each other by the authors of this study; thereafter, disagreements were discussed, and changes were made accordingly until the author team agreed that the Turkish version of the measure was ready to be finalized. Finally, the Turkish translations of the study measures were cross-checked one last time by a fourth bilingual researcher from the English instruction department and back-translated by a fifth bilingual researcher (Hambleton, 1994; van de Vijver, 2001). The entire questionnaire was also translated from Turkish into Arabic to provide an opportunity for migrant adolescents to complete the questionnaire in the language with which they were most comfortable. A professional Arabic translator produced the Arabic translation of the questionnaire (for a similar approach, see Karataş et al., 2020, 2021). The complete list of the items, both in Turkish and Arabic, is available in Supplementary Table S3.

### Results

#### Preliminary analyses

Missing value analyses indicated that missingness rates varied between 0.8% to 4.7% across items. Little (1988) MCAR test yielded a significant result, *χ*^2^ (788) = 902.079, *p* < 0.001. However, the normed *χ*^2^ (*χ*^2^/*df*) of 1.14 indicated that data were likely missing at random. As a result, all participants were included in the analyses, and missing data were handled using the FIML procedure in M*plus*. Means, standard deviations, and Cronbach’s alphas are presented in Table 2.

#### Main analyses

To test the structure validity of the ICIS in the Turkish context, we performed CFAs in M*plus* using the MLR estimator. Following the same procedure used in Study II, a solution with two latent variables and 10 observed indicators was tested. The CFA results (see Table 3) indicated that the two-factor ICIS model (i.e., positive and negative contact) fit the data well in both socialization contexts (i.e., school and out-of-school). As displayed in Figure 1B, standardized factor loadings ranged from 0.578 to 0.851 for intergroup contact interactions in the school context and from 0.632 to 0.915 for intergroup contact in out-of-school contexts (for item-total correlations, see Supplementary Table S1). Similar to the findings from Study II, negative correlations emerged between positive and negative contact in school (*r* = −0.589, *p* < 0.001) and out-of-school (*r* = −0.309, *p* < 0.001) contexts.

#### Measurement invariance

Measurement invariance tests were conducted using the same analytic procedure described in Study II to establish whether the ICIS can be applied to different ethnic groups in the Turkish context. As reported in Table 4, results indicated that both configural and metric invariance held across ethnic majority and minority adolescents. Because ΔCFI exceeded the threshold in the scalar model, ancillary analyses were conducted to identify which item intercepts might be released to obtain partial scalar invariance (Byrne et al., 1989). In this respect, we compared the scalar model with 10 other models. In each of these models, we allowed only one item intercept to vary across groups. Two sets of comparisons were carried out for the school and out-of-school forms of the ICIS across ethnic groups. Results indicated that partial scalar invariance (see Table 4) could be established by releasing intercepts of items 1, 6, 9, and 10 for intergroup contact in school context and items 1 and 9 for intergroup contact in out-of-school contexts. Thus, the ICIS can be applied with *caution* to compare the positive and negative contact of ethnic minority and majority in Turkey. Latent mean comparisons indicated that ethnic minority adolescents reported significantly higher negative contact in out-of-school contexts (*p* = 0.003, Cohen’s *d* [95% CI] = 0.33 [0.16, 0.49]) compared to Turkish adolescents.^1^

#### Convergent validity

Bivariate correlations (see Table 5) indicated that adolescents’ quantity of contact in school context was positively correlated with their positive contact in that context (*r* = 0.539, *p < 0.*001), whereas quantity of contact in school context was inversely correlated with their negative contact (*r* = −0.173, *p < 0.*001). Similarly, quantity of contact in out-of-school contexts was positively correlated with positive contact (*r* = 0.632, *p < 0.*001) and negatively correlated with negative contact (*r* = −0.083, *p < 0.*05) in this setting. As for the inter-context associations, large correlation coefficients emerged between positive contact in school and out-of-school contexts (*r* = 0.486 *p* < 0.001). Besides, large coefficients also emerged for adolescents’ negative contact across both contexts (*r* = 0.594 *p* < 0.001). Consistent with our expectations, significant positive correlations emerged between parents’ and adolescents’ positive intergroup contact (*r* = 0.234, *p < 0.*001 and *r* = 0.255, *p < 0.*001, for school and out-of-school contexts, respectively) as well as between parents’ and adolescents’ negative intergroup contact (*r* = 0.169, *p < 0.*001 for school; *r* = 0.165, *p < 0.*001 for out-of-school contexts).

#### Mean differences

Repeated measures ANOVA with two within-subject factors (i.e., contact valence and contact context) produced a significant interaction effect between the context and the valence of contact, *F* (1, 632) = 10.848, *p* < 0.01, *η*^2^ = 0.017. Ancillary pairwise comparisons showed that negative contact experiences were significantly more common in school versus out-of-school contexts. In contrast, the mean scores of adolescents’ positive contact did not significantly differ across contexts. Besides, significant differences were also found across the mean scores of adolescents’ positive contact and negative contact in both contexts, indicating that positive contact is more common than negative contact. Finally, a repeated measures ANOVA with contact quantity also demonstrated that adolescents had experienced more intergroup contact in school than in out-of-school contexts, *F* (1, 631) = 67.474, *p* < 0.001, *η*^2^ = 0.097.

### Brief discussion for Study III

In accordance with findings from Studies I and II in Italy, the CFA results demonstrated that the two-factor model, including positive and negative contact in both school and out-of-school contexts, fit the data very well. Thus, the ICIS can be administered both to ethnic majority adolescents and to ethnic minority adolescents, considering its factorial structure. However, given that equivalence of item intercepts was only partially established, comparisons between the two groups vis-à-vis their positive and negative contact should be interpreted with caution. Furthermore, as documented by a large body of evidence (e.g., Graf et al., 2014), our results also indicated more frequent positive contact in both contexts, as well as positive associations of parents’ positive and negative contact with and adolescents’ corresponding contact experiences (Bagci and Gungor, 2019). Finally, the results indicating the mean differences across school and out-of-school contexts for contact quantity but, more importantly, for negative contact also emphasized the need to consider the context in which adolescents’ intergroup contact occurs.

## General discussion

In the present set of studies, we evaluated the psychometric properties of the ICIS to assess positive and negative intergroup contact among ethnic minority and majority adolescents in school and out-of-school contexts (Study I). Thereafter, we examined the psychometric properties (i.e., internal consistency, structure and convergent validity, and ethnic measurement invariance) of the ICIS scores in both contexts across Italy (Study II) and Turkey (Study III). Results indicated that the two-factor structure of the ICIS in school and out-of-school contexts fit the data well in each cultural context. These findings are consistent with a growing body of literature suggesting that positive and negative contact are not “*polar-opposite phenomena*” (Pettigrew, 2008, p. 191); instead, they should be regarded as distinct forms of intergroup interactions (e.g., Paolini et al., 2014; Hayward et al., 2017; Barlow et al., 2019).

Another important aim of the current work was to test the ethnic invariance of the ICIS in both contexts. Except for the lack of full scalar invariance in Study III (in Turkey), the findings of the current set of studies largely suggested that the ICIS can be used to assess positive and negative intergroup contact in school and out-of-school contexts among both ethnic minority and majority adolescents in Italy and Turkey. Overall, these findings imply that we can conduct further studies within these cultural streams to advance the extant knowledge on adolescents’ positive and negative intergroup contact and their outcomes (e.g., prejudice, intergroup attitudes; Feddes et al., 2009; Titzmann et al., 2015; Wölfer et al., 2016) across school (Schachner et al., 2015) as well as out-of-school contexts such as peer groups (Albarello et al., 2021) and neighborhood (Merrilees et al., 2018).

Additionally, findings from present studies demonstrated that positive contact was more frequent than negative contact in both school and out-of-school contexts, as has been reported in the extant studies (e.g., Barlow et al., 2012; Graf et al., 2014; Hayward et al., 2017). Moreover, consistent with prior literature emphasizing the correspondence between the quantity and quality of contact (e.g., Brown et al., 2007; Jasinskaja-Lahti et al., 2011), we also found that adolescents’ perceptions concerning the quantity of their intergroup contact were positively related to positive contact in school and out-of-school contexts. Likewise, in yet opposite direction, the negative correlations between contact frequency and negative contact, specifically in the Turkish context, could also be detected. Such correlational findings provide initial evidence concerning the convergent validity of ICIS scores. Besides, the latter findings indicating adverse associations of contact frequency with adolescents’ negative contact might also be read in light of the undesirable effects of negative contact in triggering to avoid further contact. Indeed, increases in negative contact have been found to be associated with a steeper increase in avoidant tendencies (Bagci et al., 2022).

The current studies also emphasized the correspondence between parents’ and adolescents’ positive and negative contact, which fully aligns with evidence implying the intergenerational transmission (Degner and Dalege, 2013) of cross-ethnic friendships (Karataş et al., 2021). Supporting our present results, Bagci and Gungor (2019) also showed the positive links between adolescents’ positive and negative contact with their perceptions regarding the corresponding contact experiences of parents. Notably, further studies employing the ICIS would nonetheless facilitate identifying the underlying mechanisms that may account for the positive associations of parents’ positive and negative contact with adolescents’ corresponding contact experiences. Additionally, validating the ICIS for use directly with parents may also provide more accurate data on parents’ intergroup contact.

Finally, it is worth acknowledging that mean scores of adolescents’ positive contact differed between school and out-of-school contexts in Italy (Study II), whereas negative contact experiences differed between school and out-of-school contexts in Turkey (Study III). These distinct patterns within each cultural setting might be due to the relatively higher inter-ethnic tensions that mostly stem from negative perceptions towards refugees in Turkey (International Crisis Group, 2019). For example, more than half of the Turkish respondents (62.3%) in a public survey agreed with the statement indicating that Syrian refugees disrupt social morality and peace by being involved in crimes, such as violence, theft, and smuggling (Erdoğan, 2014). Inter-ethnic tensions triggered by such negative views against Syrian refugees in Turkey (as being the most represented ethnic minority group in this study) inevitably lead to more frequent negative contact experiences and their detrimental outcomes (e.g., discrimination and ostracism; Demir and Ozgul, 2019) across multiple socialization contexts. Herein, employing the ICIS in further cross-cultural studies might mirror a more nuanced picture of how perceived inter-ethnic tensions within various countries might drive such differential patterns regarding adolescents’ positive and negative contact across contexts and their essential outcomes.

## Limitations, directions for future research, and concluding remarks

The current work should be considered in light of some shortcomings. The primary limitations of the present studies stem from their cross-sectional designs, on the one hand, and the use of adolescent reports to assess parental positive and negative contact, on the other. Therefore, future longitudinal studies with multi-informant designs in which the data of parents’ contacts are directly obtained from them enable us to overcome the possible single reporter bias that might cause shared report variance. Conducting such studies by employing the ICIS would facilitate the achievement of more robust conclusions about the directionality of relationships between adolescents’ and parents’ positive and negative contact.

Another shortcoming pertains to the contexts whereby adolescents might experience both forms of contact because, in each of these studies, we examined the positive and negative contact of adolescents in one specific (i.e., school) and one broader context (i.e., out-of-school). Given that the ICIS could be easily adapted for use in other specific contexts, future studies might investigate the positive and negative forms of intergroup contact within particular out-of-school contexts such as neighborhoods, sports clubs, and peer groups to expand our understanding of adolescents’ intergroup contact. It is quite possible that the adolescents’ positive and negative intergroup contact may vary across these and other contexts.

Given that Italy and Turkey have received migrants from various ethnic groups, positive and negative contact of Italian and Turkish adolescents were measured without specifying particular ethnic groups with whom they would be in contact. However, future studies might specify particular groups in these cultural streams (e.g., Moroccans in Italy; Cicognani et al., 2018) and beyond. Given that some European countries (e.g., Poland and Germany) have recently begun to host war refugees from Ukraine (United Nations High Commissioner for Refugees, 2022), further investigating the intergroup contact in these cultural streams would expand the knowledge of the antecedents and consequences of positive and negative intergroup contact. Considering that the ICIS was designed as an instrument consisting of items assessing both adolescents’ specific intergroup interactions and their overall perspectives about positive and negative contact, the latter overall items enable assessing both forms of contact in day-to-day studies to monitor fluctuations in positive and negative contact. As such, employing the ICIS in longitudinal studies with daily assessments might also enhance a better understanding of adolescents’ positive and negative contact vis-à-vis essential intergroup outcomes, specifically in those nations and regions that host the recent Ukrainian war refugees.

Despite these and other limitations, the current set of studies provides evidence that the ICIS can be used to assess adolescents’ positive and negative intergroup contact across school and out-of-school contexts in Italy and Turkey. Besides, the present studies also indicate that the ICIS can reliably be applied to both ethnic minority and majority adolescents in these cultural settings. It is expected that the ICIS can enhance the development of further insights into the multi-faceted nature of adolescents’ intergroup contact in contemporary societies.

## Data availability statement

The datasets presented in this study can be found in online repositories. The names of the repository/repositories and accession number(s) can be found at: https://osf.io/wfhqe/?view_only=1b3da2d223e84131bf2b770bde276644.

## Ethics statement

The studies involving human participants were reviewed and approved by the ethical board of Alma Mater Studiorum University of Bologna and local authorities in Turkey. Written informed consent to participate in this study was provided by the participants' legal guardian/next of kin.

## Author contributions

SK, MR, and EC conceptualization. SK data curation and formal analysis. MR and EC funding acquisition, resources, and supervision. SK and EC investigation and methodology. SK, FP, and EC writing – original draft. MR, FP, EC, and SJS writing – review and editing. All authors contributed to the article and approved the submitted version.

## Conflict of interest

The authors declare that the research was conducted in the absence of any commercial or financial relationships that could be construed as a potential conflict of interest.

## Publisher’s note

All claims expressed in this article are solely those of the authors and do not necessarily represent those of their affiliated organizations, or those of the publisher, the editors and the reviewers. Any product that may be evaluated in this article, or claim that may be made by its manufacturer, is not guaranteed or endorsed by the publisher.

## Footnotes

^1^Given that most ethnic minority adolescents completed the Arabic version of the ICIS (*n* = 160), hierarchical levels of measurement invariance across linguistic groups (i.e., Turkish and Arabic) were also tested by following the same analytic procedure described for ethnic measurement invariance. As displayed in Supplementary Table S2, the results of the additional analyses indicated the presence of configural, metric, and partial scalar invariance for the ICIS across linguistic groups.
