# Supplementary material for: Intergroup contact in multiple adolescents’ contexts: The Intergroup Contact Interactions Scale (ICIS)
Source: Front Psychol. 2023 Jan 11;13:1066146. doi: 10.3389/fpsyg.2022.1066146 (PMC9875736; doi:10.3389/fpsyg.2022.1066146)
Supplement: Supplementary file 1 [file Table_1.docx]

**Item-Total Correlations**

**Table S1**

*Means (M), Standard Deviations (SD), and Item-Total Correlations (r) for Each Item in Studies I, II and III*

| **Items** |  | **Study I**  **(Pilot Study in Italy)** | | | **Study II**  **(Study in Italy)** | | | **Study III**  **(Study in Turkey)** | | |
| --- | --- | --- | --- | --- | --- | --- | --- | --- | --- | --- |
|  | **Contexts** | ***M*** | ***SD*** | ***r*** | ***M*** | ***SD*** | ***r*** | ***M*** | ***SD*** | ***r*** |
| 1. The experience you had with them was positive | in school | 4.065 | 0.920 | .852^***^ | 4.072 | 0.934 | .819^***^ | 3.026 | 1.186 | .726^***^ |
|  | out-of-school | 3.862 | 1.023 | .910^***^ | 3.778 | 1.120 | .891^***^ | 2.977 | 1.278 | .882^***^ |
| 2. They have been friendly toward you | in school | 4.036 | 0.953 | .937^***^ | 4.137 | 0.869 | .875^***^ | 3.190 | 1.178 | .846^***^ |
|  | out-of-school | 3.792 | 1.048 | .952^***^ | 3.857 | 1.075 | .942^***^ | 3.155 | 1.277 | .893^***^ |
| 3. They have been polite to you | in school | 4.018 | 0.934 | .909^***^ | 4.093 | 0.864 | .877^***^ | 3.173 | 1.176 | .858^***^ |
|  | out-of-school | 3.768 | 1.008 | .968^***^ | 3.866 | 1.082 | .936^***^ | 3.137 | 1.289 | .918^***^ |
| 4. They have been welcoming toward you | in school | 4.012 | 0.938 | .916^***^ | 4.003 | 0.927 | .858^***^ | 3.076 | 1.197 | .839^***^ |
|  | out-of-school | 3.775 | 1.056 | .953^***^ | 3.758 | 1.125 | .931^***^ | 3.102 | 1.280 | .916^***^ |
| 5. You felt they respected you | in school | 4.042 | 0.911 | .808^***^ | 4.051 | 0.981 | .784^***^ | 2.977 | 1.319 | .830^***^ |
|  | out-of-school | 3.783 | 1.073 | .904^***^ | 3.805 | 1.156 | .890^***^ | 3.089 | 1.367 | .877^***^ |
| 6. The experience you had with them was negative | in school | 1.708 | 0.864 | .897^***^ | 1.732 | 0.899 | .757^***^ | 2.429 | 1.209 | .698^***^ |
|  | out-of-school | 1.787 | 0.907 | .910^***^ | 1.740 | 0.924 | .825^***^ | 2.220 | 1.206 | .741^***^ |
| 7. They have been unfriendly toward you | in school | 1.687 | 0.893 | .852^***^ | 1.678 | 0.918 | .814^***^ | 2.351 | 1.206 | .801^***^ |
|  | out-of-school | 1.720 | 0.959 | .933^***^ | 1.636 | 0.911 | .862^***^ | 2.177 | 1.203 | .863^***^ |
| 8. They have been rude to you | in school | 1.704 | 0.916 | .901^***^ | 1.680 | 0.909 | .838^***^ | 2.278 | 1.241 | .828^***^ |
|  | out-of-school | 1.769 | 0.944 | .926^***^ | 1.670 | 0.932 | .877^***^ | 2.059 | 1.210 | .891^***^ |
| 9. They made you feel unwanted | in school | 1.698 | 0.968 | .863^***^ | 1.538 | 0.862 | .773^***^ | 2.435 | 1.385 | .830^***^ |
|  | out-of-school | 1.744 | 0.978 | .916^***^ | 1.534 | 0.870 | .835^***^ | 2.123 | 1.267 | .864^***^ |
| 10. They insulted you | in school | 1.620 | 0.975 | .887^***^ | 1.545 | 0.963 | .738^***^ | 1.892 | 1.263 | .790^***^ |
|  | out-of-school | 1.613 | 0.959 | .905^***^ | 1.544 | 0.919 | .825^***^ | 1.785 | 1.184 | .824^***^ |

*Note.* ^***^*p* <.001.
