# Supplementary material for: Intergroup contact in multiple adolescents’ contexts: The Intergroup Contact Interactions Scale (ICIS)
Source: Front Psychol. 2023 Jan 11;13:1066146. doi: 10.3389/fpsyg.2022.1066146 (PMC9875736; doi:10.3389/fpsyg.2022.1066146)
Supplement: Supplementary file 2 [file Table_2.docx]

**Linguistic Measurement Invariance Tests**

In addition to ethnic majority adolescents (i.e., Turkish, *n* = 430), 51 ethnic minority adolescents completed the ICIS in Turkish. Among these ethnic minority youth, 151 adolescents completed the scale only in Arabic, and 9 adolescents filled the scale in both Turkish and Arabic. This latter group of adolescents (*n* = 9) was combined with those who filled in the scale in Arabic by considering their reports regarding which language they prefer to speak most of the time with their interactions with parents (*n*_equally in Turkish and Arabic_ = 3; *n*_mostly in Arabic_ = 1; *n*_only in Arabic_ = 5) and friends (*n_mostly in Turkish_* = 1; *n*_equally in Turkish and Arabic_ = 5; *n*_mostly in Arabic_ = 2; *n*_only in Arabic_ = 1). Consequently, linguistic measurement invariance of the ICIS in both contexts was tested across the groups of adolescents who completed the scale in Turkish (*n* = 481) or Arabic (*n* = 160).

Similar to the ethnic measurement invariance findings, the current results showed that configural, metric, and partial scalar invariance held across linguistic groups. Hence, the ICIS can be applied with *caution* to compare the positive and negative contact of adolescents who completed the scale in Turkish and those who completed it in Arabic.

**Table S2**

*Results of the Linguistic Measurement Invariance Tests of the ICIS in School and Out-of-School Contexts in Study III*

|  | **Model fit indices** | | | | | | **Model comparison** | | | | | |
| --- | --- | --- | --- | --- | --- | --- | --- | --- | --- | --- | --- | --- |
|  | ***χ*_SB_^2^** | ***df*** | **CFI** | **TLI** | **SRMR** | **RMSEA**  **[90% CI]** | **Models** | **Δχ_SB_^2^** | **Δ*df*** | ***p*** | **ΔCFI** | **ΔRMSEA** |
| Linguistic Invariance of the ICIS in the School Context | | | | | | |  |  |  |  |  |  |
| M1. Configural model | 203.188 | 68 | .943 | .924 | .042 | .079 [.067, .092] |  |  |  |  |  |  |
| M2. Metric model | 215.338 | 76 | .941 | .930 | .050 | .076 [.064, .088] | M2-M1 | 10.126 | 8 | .256 | -.002 | -.003 |
| M3. Scalar model | 283.184 | 84 | .916 | .910 | .057 | .086 [.075, .098] | M3-M2 | 73.612 | 8 | .000 | -.025 | .010 |
| M3a. Partial scalar model^a^ | 239.485 | 80 | .933 | .924 | .049 | .079 [.068, .091] | M3a-M2 | 25.653 | 4 | .000 | -.008 | .003 |
| Linguistic Invariance of the ICIS in the Out-of-School Context | | | | | | | |  |  |  |  |  |
| M1. Configural model | 156.317 | 68 | .969 | .959 | .028 | .064 [.051, .077] |  |  |  |  |  |  |
| M2. Metric model | 180.573 | 76 | .964 | .957 | .044 | .066 [.053, .078] | M2-M1 | 26.733 | 8 | .001 | -.005 | .002 |
| M3. Scalar model | 230.782 | 84 | .949 | .945 | .052 | .074 [.063, .086] | M3-M2 | 58.568 | 8 | .000 | -.015 | .008 |
| M3a. Partial scalar model^a^ | 208.622 | 82 | .956 | .952 | .051 | .070 [.058, .081] | M3a-M2 | 31.990 | 6 | .000 | -.008 | .004 |

*Note.* χ_SB_^2^ = Satorra-Bentler scaled chi-square; *df* = degrees of freedom; CFI = Comparative Fit Index; TLI = Tucker-Lewis Index; SRMR = Standardized Root Mean Square Residual; RMSEA [90% CI] = Root Mean Square Error of Approximation and 90% Confidence Interval; Δ = Change in the parameter.

^a^ Partial scalar invariance across linguistic groups (i.e., Turkish and Arabic) could be established by releasing intercepts of items 2, 4, 6, and 9 for intergroup contact in school context and intercepts of items 1 and 9 for intergroup contact in out-of-school contexts.
