# Supplementary material for: Intergroup contact in multiple adolescents’ contexts: The Intergroup Contact Interactions Scale (ICIS)
Source: Front Psychol. 2023 Jan 11;13:1066146. doi: 10.3389/fpsyg.2022.1066146 (PMC9875736; doi:10.3389/fpsyg.2022.1066146)
Supplement: Supplementary file 3 [file Table_3.docx]

**Table S3**

*Completed List of Items of the ICIS in Each Language*

**Prompt:** *“*The following questions are about interactions you may have had in school [out-of-school] context with people of foreign origin [Italian/Turkish people]”.

Now think about the interactions you had in the school (for example with other students, with teachers) [out-of-school (for example in your neighborhood, in your sports club)].

| **Item Number** | **English** | **Italian** | **Turkish** | **Arabic** | |
| --- | --- | --- | --- | --- | --- |
|  | *In the past six months at school (out-of-school)…* | *Negli ultimi sei mesi, a scuola (fuori dalla scuola)…* | *Son altı ayda, okulda (okul dışında)...* | | في الأشهر الستة الأخيرة في المدرسة (خارج المدرسة)... |
| **Positive Contact** | |  |  | |  |
| Item 1 | The experience you had with them was positive. | L’esperienza che hai avuto con loro è stata positiva. | Onlarla yaşadığın deneyim olumluydu. | | كانت التجربة التي عشتَها أنت معهم ايجابية. |
| Item 2 | They have been friendly toward you. | Sono stati amichevoli nei tuoi confronti. | Sana karşı arkadaşçaydılar. | | كانوا ودودين تجاهك. |
| Item 3 | They have been polite to you. | Sono stati gentili con te. | Sana karşı naziktiler. | | كانوا لطفاء معك. |
| Item 4 | They have been welcoming toward you. | Sono stati accoglienti nei tuoi confronti. | Sana karşı kabul edici davrandılar. | | تصرفوا معك بشكل مقبول. |
| Item 5 | You felt they respected you. | Ti sei sentito/a rispettato/a. | Sana saygı duyduklarını hissettin. | | شعرت بأحترامهم لك. |
| **Negative Contact** | | | | | |
| Item 6 | The experience you had with them was negative. | L'esperienza è stata negativa. | Onlarla yaşadığın deneyim olumsuzdu. | كانت التجربة التي عشتَها أنت معهم سلبية. | |
| Item 7 | They have been unfriendly toward you. | Sono stati ostili nei tuoi confronti. | Sana karşı arkadaşça değildiler. | لم يكونوا ودودين تجاهك. | |
| Item 8 | They have been rude to you. | Sono stati maleducati con te. | Sana karşı kabaydılar. | كانوا وقحين معك. | |
| Item 9 | They made you feel unwanted. | Ti hanno fatto sentire non voluto/a. | Sana istenmediğini hissettirdiler. | جعلوك تشعر بأنك غير مرحب به. | |
| Item 10 | They insulted you. | Ti hanno insultato/a. | Seni aşağıladılar. | اهانوك. | |
| Response Scale | 1 = Never  2 = Rarely  3 = Sometimes  4 = Often  5 = Very often | 1 = Mai  2 = Raramente  3 = Qualche volta  4 = Spesso  5 = Molto spesso | 1 = Hiçbir zaman  2 = Nadiren  3 = Bazen  4 = Sıklıkla  5 = Çok sıklıkla | 1 = ولا مرة  2 = نادر جداً  3 = احياناً  4 = على الاكثر  5 = دائماً | |
